# Quantity of documentation of maltreatment risk factors in injury-related paediatric hospitalisations

**DOI:** 10.1186/1471-2458-12-563

**Published:** 2012-07-28

**Authors:** Kirsten McKenzie, Debbie A Scott

**Affiliations:** 1 Centre for Accident Research and Road Safety Queensland, Queensland University of Technology, Victoria Park Road, Kelvin Grove, QLD 4059, Australia; 2 Australian Institute of Family Studies, Latrobe St, Melbourne, Victoria 3000, Australia

**Keywords:** Child maltreatment, Clinical documentation, Data linkage, Injury surveillance, Morbidity data

## Abstract

**Background:**

While child maltreatment is recognised as a global problem, solid epidemiological data on the prevalence of child maltreatment and risk factors associated with child maltreatment is lacking in Australia and internationally. There have been recent calls for action to improve the evidence-base capturing and describing child abuse, particularly those data captured within the health sector. This paper describes the quantity of documentation of maltreatment risk factors in injury-related paediatric hospitalisations in Queensland, Australia.

**Methods:**

This study involved a retrospective medical record review, text extraction and coding methodology to assess the quantity of documentation of risk factors and the subsequent utility of data in hospital records for describing child maltreatment and data linkage to Child Protection Service (CPS).

**Results:**

There were 433 children in the maltreatment group and 462 in the unintentional injury group for whom medical records could be reviewed. Almost 93% of the maltreatment code sample, but only 11% of the unintentional injury sample had documentation identified indicating the presence of any of 20 risk factors. In the maltreatment group the most commonly documented risk factor was history of abuse (41%). In those with an unintentional injury, the most commonly documented risk factor was alcohol abuse of the child or family (3%). More than 93% of the maltreatment sample also linked to a child protection record. Of concern are the 16% of those children who linked to child protection who did not have documented risk factors in the medical record.

**Conclusion:**

Given the importance of the medical record as a source of information about children presenting to hospital for treatment and as a potential source of evidence for legal action the lack of documentation is of concern. The details surrounding the injury admission and consideration of any maltreatment related risk factors, both identifying their presence and ruling them out are required for each and every case. This highlights the need for additional training for clinicians to understand the importance of their documentation in child injury cases.

## Background

While child maltreatment is recognised as a global problem, solid epidemiological data on the prevalence of child maltreatment and risk factors associated with child maltreatment is lacking in Australia and internationally. There have been recent calls for action to improve the evidence-base capturing and describing child abuse, particularly those data captured within the health sector [1,2]. Data from the USA has shown that 10% of all children presenting to an Emergency Department are victims of child abuse and neglect and without identification, 35% will be injured again and 5% will die from subsequent injuries [3].

Nationally and internationally, hospital morbidity data are key standardised indicators used to assess population health, with these data coded according to the International Statistical Classification of Diseases and Related Health Problems (ICD) system developed by the World Health Organisation [4]. However, these data which are used for public health research and policy development, rely on detailed clinical documentation in hospital records by health professionals [5,6]. Medical records are an important source of information and communication between clinical staff, and the primary source document for routine clinical coding of morbidity. Limited clinical documentation around suspicions of child maltreatment can significantly hamper the process of identification and coding of such cases. Furthermore, comprehensive documentation in cases of suspected maltreatment is critical to facilitate adequate response and follow-up, both clinically and legally [7]. While a complete, concise clinical description of a maltreated child does not exist, there are well accepted clinical indicators that are suspicious of abuse to assist clinicians in the identification process when a child presents for treatment [8]. Taitz et al (2004) found that inconsistent histories, fractures, particularly in children under 1 year and certain fracture types deemed high-risk (e.g. bucket handle) and multiple presentations for fractures, as well as presentation delays, and a lack of witnesses to injury events [9] were all potential indicators of physical abuse in children.

Furthermore, it could be argued that a completely documented assessment for all child injury presentations is necessary to minimise the risk of missing potential child maltreatment related injury. Zeigler et al found that in Emergency Department (ED) records, only 20% of cases of fractures in children under three years of age had documentation to indicate whether child maltreatment had been considered by the emergency physician, and 27% of cases had inadequate documentation to assess the consistency of the injury with the case history [10]. Limbos et al found in those cases discharged with a diagnosis of child maltreatment, only 45% of medical records had documentation specifying the history of injuries and over a quarter of cases omitted a description of the physical examination findings [11]. Limbos et al concluded that the majority of records contained “inadequate data to determine retrospectively if the diagnosis of child maltreatment had ever been considered by the evaluator” [11]. Boyce et al (1996) reported that only one-third of paediatric trauma cases had sufficient documentation to identify potential abuse from medical records [12].

Newton et al (2010) undertook a systematic review of interventions aimed at improving documentation and clinical assessment where suspicions of child abuse were present. They identified only six studies which reported such interventions, four of which focused on assessing improvements in documentation quality. While there was consensus across all of these around documentation quality, two in particular surmised that while there were some studies identifying improvements in documentation after education and reminder systems were implemented [13,14], the lack of high quality studies to validate these findings limited the strength of these conclusions. Guenther et al (2009) attempted to improve the documentation of child maltreatment indicators via an educational intervention with emergency department physicians, but found no significant difference in documentation patterns pre and post intervention [15]. Limbos and Berkowitz (1998) evaluated the quality of documentation in 1995 compared to 1980 for children diagnosed as abused, and reported that the only indicators which improved marginally were documentation of involvement of child protection (77% of cases in 1980 up to 100% of cases in 1995) and the disposition of cases (66% of cases in 1980 up to 90% of cases in 1995) [11].

The identification of child maltreatment injuries in routine health data collections, such as hospital morbidity data collections, enables an efficient and nationally standardised source to capture valid and reliable health data and information pertaining to the circumstances surrounding hospitalisation. However, to date, there has been no research conducted which examines the collection and quantity of child maltreatment information in routinely collected health data collections in the Australian context and only limited research at an international level. The current study examined the quantity of documentation of maltreatment risk factors in injury-related paediatric hospitalisations in Queensland, Australia.

## Methods

This study involved a retrospective medical record review, text extraction and coding methodology to assess the quantity of documentation of risk factors and the subsequent utility of data in hospital records for describing child maltreatment and data linkage to Child Protection Service (CPS)*.* A stratified sample of hospitals across Queensland was selected for this study, and from within those hospitals a sample of cases were selected for review. A previous article written by the authors has described the hospital and patient selection in detail and as such, this article will only describe this process briefly [6].

### Sample selection

The aim in selecting hospitals in the sample was to identify a representative sample of large, medium and small caseload public hospitals from metropolitan, rural and remote areas throughout Queensland. The final sample of 20 hospitals included (7 large, 7 medium, and 6 small). Once hospitals were sampled, ethical clearances were obtained from all relevant hospital ethics committees to seek approval to access medical records from each facility. Once ethical approvals were obtained, a second stage of sampling was undertaken to identify patients for inclusion in the study.

Sample selection of patients was conducted using the State Health Department admitted patient data collection (Queensland Health Admitted Patient Collection (QHADPC)), which contains unit record data for each hospital separation from all hospitals within the State. The admission year range for selection of cases was 2003 to 2006, and the age range included only cases under 18 years of age. To ensure a sufficient sample of cases of child maltreatment in the “Any Maltreatment Code” sample, cases were eligible for selection if there was a range of definitive maltreatment codes or possible maltreatment codes assigned as either a principal diagnosis, an additional diagnosis, an external cause or a procedure in the patient’s hospital separation data as follows (with each of these codes present for at least one or more sampled cases):

a) One or more of the ICD-10-AM codes T74.0-T74.9, Z04.4, Z04.5, Z61.4, Z61.5, Z61.6, Z62.0, Z62.3, Z62.4, Z62.5, Z62.6

b) An ICD-10-AM external cause code in the range X85-Y09 with a fifth-digit value of 1, 2, or 3 for 15 to 17 year old children or a fifth-digit value of 1, 2, 3, 8, or 9 for patients under 15 years of age, or

c) An ACHI procedure code of 5830600 or 9608400 reported in any of the procedure codes assigned.

To take into account the different caseloads of different hospitals and to approximate a probability-based result, the number of cases for selection at each hospital was stratified according to the size of the hospital. The initial screening of QHADPC data found 511 eligible cases available for review that had any of the above codes assigned, and all of these cases were included in the final sample. In addition, a random sample of 500 cases coded as being due to an unintentional injury (i.e. ICD-10-AM external cause codes in the ‘Accident’ code range V00-X59, with no codes used in the maltreatment/possible maltreatment code range, nor codes from the assault (X85-Y09) or undetermined intent code ranges (Y10-Y34) in the ICD-10-AM) were extracted as a comparison group, and to explore documentation where the presence of risk factors were ruled out. These were grouped into an “Unintentional Injury Code” sample.

### Data collection process

Inclusion and exclusion criteria for the targeted sample were provided to the State Health department, and the department extracted the unit record numbers (URN) and hospital identifiers of the sample of records. The department then provided lists of URNs to Health Information Managers (HIMs) within each hospital who extracted individual medical records for the research team to access on site. Two members of the research visited each site and reviewed the medical records. The researcher reviewed the records and extracted text excerpts from the medical records to describe the documented circumstances surrounding the injury event for all cases. Information was extracted from each of the following documentation sources if present: Emergency Department Notes, Admission Notes, Discharge Summary, and the Queensland Health Suspected Child Abuse and Neglect report.

A data collection database was designed for the capture of data and a data collection manual specified the aims of the project, the process for data collection, and items to be collected, and provided a detailed description of the data collection database. The data collection manual prompted researchers to collect all relevant information about circumstances surrounding the event, place of occurrence and activity at time of the event, as well as documented risk factors. Excerpts from the Queensland Health training manual for Child Abuse and Neglect education [16] and from the International Classification of External Causes of Injury manual [17] were provided in the data collection manual to highlight which relevant risk indicators should be extracted and documented. All researchers were provided both off-site and on-site training in the use of the database and the process for collection of data.

### Coding of text extracts

Text extracts were reviewed by researchers (KM and DS) and an additional summary form was created in the database with tickbox fields to indicate the presence or absence of text to describe each of the following risk factors:

1. History - History of abuse, History of foster care, Previous admissions to hospital, Known to the Child Safety Department

2. Health/behavioural- Poor physical appearance, Behavioural cues indicative of abuse, Poor general health

3. Protective- Lack of a protective parent/adult

4. Substance abuse-Drug abuse of the child/family, Alcohol abuse of the child/family

5. Disabilities-Physical disabilities of the child/family, Intellectual disabilities of the child/family, Mental health history of the child/family

6. Socioeconomic-Poor socioeconomic circumstances, Homelessness of the child/family, Transient living circumstances

7. Criminal-Criminal history of the child/family, Police involvement with the child/family

8. Family relationship – Relationship instabilities in the family, Domestic violence in the family.

The risk indicators were coded with a value of 1 if it indicated a heightened risk for the child (eg. A history of abuse documented) and a value of 0 if it did not represent a heightened risk for the child (eg. No history of abuse documented). A variable was created which added the values for each of risk indicator variables to provide a sum of all documented risk indicators (with a maximum score of 20 given the 20 risk indicators which were coded).

### Data linkage

A dataset containing the researchers’ project ID and identifying details of the child was provided by the health department to CPS. An experienced CPS client intake officer manually linked the data using routine CPS intake procedures for each record. De-identified data were then provided to the researchers and these data were merged with the de-identified health dataset using the project ID.

### Data analysis

PASW Version 18 was used to conduct descriptive analyses, using frequencies and percentages to quantify the numbers and proportions of patients with documented risk factors for the any maltreatment code sample and the unintentional injury code sample.

### Ethics

Ethics approval to conduct this analysis was obtained from the Queensland Health Human Research Ethics Committee, the Queensland University of Technology Ethics Committee, and the human research ethics committees at each hospital site where data was collected (names withheld to protect the confidentiality of the data collection).

## Results

### Sample characteristics

There were 433 unique children in the maltreatment code sample and 462 children in the unintentional injury code sample for which data could be reviewed. The remaining cases were unable to be included in the study as their medical records were unavailable at the point of data collection at the hospitals, with 90% of the maltreatment code sample available for review and 95% of the unintentional injury code sample available for review (Note: there was no significant difference between available and unavailable cases in terms of gender, locality, or linkage rates to child protection, but children whose records were unavailable were marginally older than those whose records were available with an average age of 9.6 (SD 5.8) for unavailable cases and 8.3 (SD 5.8) for available cases). Table 1 shows age and sex distributions for each code group. There were differences in the age and sex distribution by code group, with a larger number of females than males in the any maltreatment code group. Almost 64% of the sample with a maltreatment code were female, while females comprised only 34% of the unintentional injury sample. Age distributions varied in each code group by gender with the largest proportion of males with any maltreatment code in the <1 year age group (31%) and the 1-5 year age group (30%), while for females with any maltreatment code the largest proportion were in the 10-14 year age group (30%) followed by the 1-5 year age group (26%) (varying substantially from the patterns in the unintentional injury code group).

**Table 1:** Age Groups and Sex Distribution of Children by Code Group

**Sex and age groups**	**Unintentional injury code**		**Any maltreatment code**		**Total**	
	**n**	**Col%**	**n**	**Col%**	**n**	**Col%**
Males						
<1	9	2.9	49	31.4	58	12.6
1-5	69	22.5	46	29.5	115	24.9
6-9	57	18.6	17	10.9	74	16.0
10-14	94	30.7	34	21.8	128	27.7
15-17	77	25.2	10	6.4	87	18.8
Total	306	100.0	156	100.0	462	100.0
Females						
<1	9	5.8	39	14.1	48	11.1
1-5	51	32.7	71	25.6	122	28.2
6-9	39	25.0	28	10.1	67	15.5
10-14	29	18.6	82	29.6	111	25.6
15-17	28	17.9	57	20.6	85	19.6
Total	156	100.0	277	100.0	433	100.0
Total						
<1	18	3.9	88	20.3	106	11.8
1-5	120	26.0	117	27.0	237	26.5
6-9	96	20.8	45	10.4	141	15.8
10-14	123	26.6	116	26.8	239	26.7
15-17	105	22.7	67	15.5	172	19.2
Total	462	100.0	433	100.0	895	100.0

### Documented risk factors

Almost 93% of the any maltreatment code sample but only 11% of the unintentional injury code sample had documentation identified which indicated the presence of any of the 20 risk factors which were investigated. The average number of risk factors documented for the any maltreatment code sample was 3.3 (95% CI 3.1-3.5), with a maximum number of risk factors for any one child of 12 risk factors. The average number of risk factors documented for the unintentional injury code sample was 0.2 (95% CI 0.13-0.26), with a maximum number of risk factors for any one child of 5 risk factors. Table 2 lists the number and percentage of cases with documentation of each of the risk factors (ordered by most frequently documented risk factors for the any maltreatment code group). The most commonly documented risk factors for the maltreatment code group were a history of abuse (41%), being known to the child safety department (41%), and a mental health history for the child or family (40%). Few unintentional injury cases had documented risk factors, but the most commonly documented risk factor in this group was alcohol abuse of the child or family (3%).

**Table 2:** Risk Factor Documentation by Code Group

**Risk factor**	**Unintentional Injury Code**		**Any Maltreatment Code**	
	**n**	**%**	**n**	**%**
History of abuse	7	1.5	179	41.3
Known to the Child Safety Department	10	2.2	178	41.1
Mental health history of the child/family	10	2.2	175	40.4
Police involvement with child and/or family	5	1.1	125	28.9
Alcohol abuse of the child/family	15	3.2	95	21.9
Drug abuse of the child/family	9	1.9	94	21.7
Domestic violence in family	3	0.6	90	20.8
Poor general health of child	4	0.9	89	20.6
Relationship instabilities in family	4	0.9	68	15.7
History of foster care	2	0.4	60	13.9
Previous admissions of child	9	1.9	46	10.6
Poor physical appearance	1	0.2	45	10.4
Criminal history of the child/family	3	0.6	43	9.9
Behavioural cues indicative of abuse	1	0.2	31	7.2
Transient living circumstances	1	0.2	31	7.2
Poor socioecomonic circumstances	3	0.6	28	6.5
Homelessness of the child/family	0	0.0	25	5.8
Lack of protective parent/adult	1	0.2	19	4.4
Intellectual disabilities of the child/family	1	0.2	19	4.4
Physical disabilities of the child/family	1	0.2	6	1.4

### Documentation of risk factors by demographics

Documentation of risk factors differed across age and gender for the different code groups (See Table 3). In the unintentional injury code group, males <1 year old had the highest documentation of risk factors (44% had at least one risk factor documented), and females in the 15-17 year age group had the highest documentation of risk factors (36% had at least one risk factor documented). Comparing those cases in the unintentional injury code group with any documentation of risk with those cases in the unintentional injury code group with no documentation of risk, the only factor found to vary significantly was age group, with children aged 1-14 significantly less likely to have documented risk factors than children aged under 1 year of age (variables included in binary logistic regression were age group, gender, hospital size and locality, injury type and external cause mechanism). For the any maltreatment code sample, all male children 6 years of age and older had at least one documented risk factor, while females 15-17 year olds had the highest documentation of risk factors (98%). Small cell sizes prevented a multivariate analysis of contributory factors for the maltreatment code sample.

**Table 3:** Risk Factors Documentation by Age Groups and Sex Distribution of Children by Code Group

**Sex and Age Groups**	**Unintentional Injury Code**			**Any Maltreatment Code**		
	**Mean**	**SD**	**% with any risk documented**	**Mean**	**SD**	**% with any risk documented**
Males						
<1	.67	1.00	44.4	3.00	2.13	85.7
1-5	.10	.43	7.2	2.61	1.72	89.1
6-9	.05	.23	5.3	2.71	1.61	100.0
10-14	.19	.69	10.6	3.74	1.64	100.0
15-17	.23	.65	14.3	4.50	2.01	100.0
Total	.17	.58	10.8	3.11	1.91	92.3
Females						
<1	.22	.44	22.2	2.79	1.88	89.7
1-5	.25	1.02	7.8	2.82	1.91	90.1
6-9	.03	.16	2.6	3.11	1.77	96.4
10-14	.03	.19	3.4	3.90	2.19	92.7
15-17	.75	1.29	35.7	4.30	2.35	98.2
Total	.24	.85	11.5	3.47	2.15	93.1
Total						
<1	.44	.78	33.3	2.91	2.02	87.5
1-5	.17	.74	7.5	2.74	1.83	89.7
6-9	.04	.20	4.2	2.96	1.71	97.8
10-14	.15	.61	8.9	3.85	2.04	94.8
15-17	.37	.89	20.0	4.33	2.29	98.5
Total	.19	.68	11.0	3.34	2.07	92.8

### Documentation of risk factors by child protection system involvement

Over 93% of the maltreatment code sample and 32% of the unintentional injury code sample were able to be linked to a record in the child protection system data. Of the 7% in the maltreatment code sample that didn’t link to a child protection system record, 17% had no documented risk factors. However, of the 93% in the maltreatment code sample that *did* link to a child protection system record, only 6% had no documented risk factors. By comparison, of the 68% in the unintentional injury code sample that didn’t link to a child protection system record, 93% had no documented risk factors and of the 32% in the unintentional injury code sample that *did* link to a child protection system record, 80% had no documented risk factors. Table 4 shows the percentage of cases in each age/sex group with and without documented risk factors for the cases that linked to a record in the child protection system data.

**Table 4:** Frequency and Percent of Risk Factor Documentation for Linked CPS Cases by Age and Sex by Code Group

**Sex and Age Groups**	**Unintentional Injury Code**		**Any Maltreatment Code**	
	**n**	**%**	**n**	**%**
Males				
<1	2	50.0	42	89.4
1-5	5	23.8	39	90.7
6-9	0	0.0	17	100.0
10-14	7	21.2	31	100.0
15-17	6	21.4	9	100.0
Total	20	20.2	138	93.9
Females				
<1	1	100.0	35	92.1
1-5	2	10.5	62	91.2
6-9	0	0.0	25	96.2
10-14	0	0.0	70	92.1
15-17	6	66.7	48	98.0
Total	9	18.0	240	93.4
Total				
<1	3	60.0	77	90.6
1-5	7	17.5	101	91.0
6-9	0	0.0	42	97.7
10-14	7	17.5	101	94.4
15-17	12	32.4	57	98.3
Total	29	19.5	378	93.6

Furthermore, examining the child protection system records for children with and without documented risk factors in both the unintentional injury code sample and the maltreatment code sample revealed different patterns. While almost 24% of children in the unintentional injury code sample with one or more risk factors documented were known to the child protection system during the hospital admission or became known within 12 months of discharge, only 7% of children in the unintentional injury code sample with no risk factors documented were known to the child protection system in the same time frame (See Table 5). In contrast, almost half of the children with a maltreatment code had a current child protection system record, regardless of the presence/absence of risk factor documentation, with 31% of those with risk factor documentation having a child protection record activated within 12 months of discharge, and 23% of those without risk factor documentation having a child protection record activated in the same time frame.

**Table 5:** Risk Factor Documentation by Code Group by Time to Next Child Protection Report

**Length of time to next CPS report**	**Unintentional Injury Code**				**Any Maltreatment Code**			
	**0 risk factors**		**>=1 risk factor**		**0 risk factors**		**>=1 risk factor**	
	**n**	**%**	**n**	**%**	**n**	**%**	**n**	**%**
Not known to CPS	291	70.8	22	43.1	5	16.1	24	6.0
Current CPS event	2	0.5	2	3.9	15	48.4	194	48.3
Between discharge-12 months	26	6.3	10	19.6	7	22.6	126	31.3
Between 1-3 years	24	5.8	8	15.7	2	6.4	26	6.5
>3 years after discharge	68	16.5	9	17.6	2	6.4	32	8.0
Total	411	100	51	100	31	100	402	100

The average number of risk factors documented for the any maltreatment code sample that linked to a child protection system record was 3.4 (SD 2.1), and the average number of risk factors documented for the any maltreatment code sample that didn’t linked to a child protection system record was 2.2 (SD 1.7). The average number of risk factors documented for the unintentional injury code sample that linked to a child protection system record was 0.4 (SD 0.9), and the average number of risk factors documented for the unintentional injury code sample that didn’t link to a child protection system record was 0.1 (SD 0.5).

While small cell sizes prevented multivariate analysis, descriptive analysis of the unintentional injury code sample who had at least one risk factor documented showed that the highest proportions of children that linked to a child protection record were aged 2-5 years (78% linkage), were male (61% linkage), presented at small hospitals (83% linkage), presented at rural/remote hospitals (68% linkage), were poisoning-related presentations (80% linkage) (Note: the percentage that linked overall for unintentional injury cases with at least one risk factor documented was 57%). Very small cell sizes prevented any descriptive analysis of contributory factors for the maltreatment code sample with documented risk factors.

## Discussion

This study assessed the quantity of documentation of maltreatment risk factors in injury-related paediatric hospitalisations for two samples of patients, those with a discharge diagnosis indicating definitive or possible child maltreatment and those with a discharge diagnosis of an unintentional injury. The assignment of a maltreatment code relies on documentation in the medical record to indicate that maltreatment is a possibility, and hence it is not surprising that 93% of cases with a maltreatment code had some documented risk factors in the medical record. For the maltreatment code sample, the most commonly documented risk factor was a history of abuse (45%), and other research has found a similar proportion of documentation of abuse history for maltreatment coded cases (41%) [11]. Being known to the child protection department and the presence of mental health issues in the child or family were documented almost as frequently as the presence of a history of abuse. Parental mental illness, particularly maternal illness has been associated with a higher risk of child maltreatment in previous research [18].

Examining the extent of documentation of risk factors in relation to their child protection system status offered an external validation of likely child maltreatment (given that contact with the child protection system is generally due to concerns about the risk of maltreatment). Over 93% of the maltreatment code sample were able to be linked with a record on the child protection system for either previous, current or subsequent maltreatment concerns, though 16% of those that linked to child protection had no documented risk factors in the medical record. Given that these cases were coded as maltreatment related and the broad scope of factors included in the risk factor review this absence of documented risk factors may reflect poor documentation practises.

In contrast, only 11% of cases in the unintentional injury code sample had a risk factor documented in the medical record, despite 32% of the unintentional injury code sample linking with a record on the child protection system. Only 20% of those that linked had one or more risk factors documented in the medical record. Within the unintentional injury code sample, males under 1 year of age were the most likely group to have documentation of risk factors with 44% of cases having some risk factor documented and the most commonly documented risk factor was previous injury-related hospital admission. Children under 12 months are the most vulnerable age group for child maltreatment and epidemiological research has found they frequently comprise the largest proportion of children with abusive injury [19]. The second most common group to have documentation of risk factors was females aged 15-17 years of age. The most common risk factors documented in this group were alcohol or drug abuse, mental health issues or relationship instabilities. Research has demonstrated that adolescent girls who have been maltreated are at increased risk of illicit drug or alcohol abuse, self harm, including suicide and mental health issues, including depression [20]. While many of these presentations may have been due to a legitimate unintentional cause of injury, the lack of documentation indicating the consideration of risk factors associated with maltreatment raises concerns that injuries due to maltreatment are not being identified. Given that a higher proportion of children with an unintentional injury code with risk factors documented than without risk factors documented linked to a child protection record in the 12 months post discharge, risk factor documentation in medical records may be used as an early warning of potential harm for children otherwise treated for injuries deemed to be unintentional.

Providing a definitive diagnosis for injury and whether or not it is related to maltreatment can be difficult for clinicians, particularly where parents cannot or will not provide information about the injury event or the information they provide differs from what occurred to cause the injury. The clinician is required to assess the injury and determine whether or not the injury occurred due to a lapse in supervision that may or may not be associated with supervisory neglect, or if a parent or caregiver has deliberately inflicted the injury. Research has shown that parents may provide false information about the reason for the child’s presentation to hospital [21] and relying completely on that information could result in missing some children who are being maltreated. This serves to reinforce the importance of complete documentation in the medical record. Where the documentation is complete and describes all elements considered in arriving at a diagnosis of maltreatment, or in ruling it out, previous admissions can be referred to and assist in identifying patterns of concerning behaviour or circumstances that can assist in decision making, diagnoses and, where necessary, reporting of maltreatment. Furthermore, mandatory reporting legislation in this state require the reporting of maltreatment based on a reasonable suspicion that harm is occurring, has occurred or may occur in the future [22] – therefore the documentation required to demonstrate that maltreatment was considered and, where suspected ruled out, is important should the record ever be required in court for legal purposes.

This study does have some limitations. Documentation of risk factors is not a validated measure of existence of these risk factors, but merely an approximation of risk. With limited guidance for clinical staff regarding what factors require documentation in circumstances of maltreatment, documented risk factors are a proxy measure at best. However, it is still important to examine the extent of documentation and the concordance of documentation with child protection records to gauge the likely completeness of documentation practises. While this study has shown some consistent estimates (such as 93% of cases with maltreatment codes having documented risk factors, and a similar proportion of these cases being known to child protection), there were some divergences in the estimates (such as only 11% of the unintentional injury cases having documented risk factors despite almost one-third of this group having a child protection record). Secondly a lack of documentation may not indicate a failure to consider the presence of maltreatment related risk factors, the clinician may simply not have documented this in the medical record. However, this is, of itself, problematic given the importance of the medical record for communication between health staff and its potential use as a source of evidence for prosecution of perpetrators of maltreatment or information for future admissions that may be suspicious for maltreatment.

A potential limitation of this study could be the use of ICD codes to identify maltreatment in the administrative data sets that formed the basis of the study sample. Some research has shown that ICD is likely to under-identify maltreatment if only the definitive codes for abuse are used [23,24]. In this research a broad range of ICD codes that included those identified as associated with possible abuse and a sample not associated with abuse were included so the under-representation is likely to be less of an issue than if only definitive codes were used. The research is still likely to under-represent neglect and other forms of maltreatment that are not associated with physical injury in children.

Ethics committees denied researchers the right to collect and analyse data based on the Indigenous status of the child. In Australia, Indigenous children are over-represented in all child protection statistics from notifications through substantiations and out of home care [19]. This is likely to be important in considering the risk factors associated with maltreatment in this study as well however, the focus of these analyses were the documentation in the medical record. While it is unlikely that documentation would have varied by Indigenous status no analyses were possible to see if there was a variation in documentation according to the Indigenous status of the child.

## Conclusion

The apparent lack of documentation for a proportion of cases suggests that clinicians may require additional training about the importance of thorough documentation for all paediatric injury/maltreatment-related hospitalisations. The details surrounding the injury admission and consideration of any maltreatment related risk factors, both identifying their presence and ruling them out are required for each and every case. Further research is needed to explore the nature of documentation for different causes and types of injury, and across different regions/hospitals to gain a more in-depth understanding of documentation patterns. Furthermore, intervention efficacy studies to improve and evaluate documentation practices are needed in an Australian context.

## Competing interests

The authors declare that they have no competing interests.

## Authors’ contribution

Both authors made an important contribution to the development of the research concept and methodology, data collection, data entry and interpretation of results. KM conducted all analyses for this manuscript. KM was primarily responsible for developing the first draft of the manuscript, and DS refined and completed the draft. Both authors read and approved the final manuscript.

## Pre-publication history

The pre-publication history for this paper can be accessed here:

http://www.biomedcentral.com/1471-2458/12/563/prepub
